# Dietary Antigen Interaction with Intestinal Epithelial Cells

**DOI:** 10.1007/s11882-026-01254-9

**Published:** 2026-02-19

**Authors:** Paula E. Reichel, Somdutta Chakraborty, Hock L. Tay, Simon P. Hogan

**Affiliations:** 1 Mary H. Weiser Food Allergy Center, Michigan Medicine, University of Michigan, 109 Zina Pitcher Place, Ann Arbor, MI 48109-2200 USA; 2 Department of Pathology, Michigan Medicine, University of Michigan, 109 Zina Pitcher Place, Ann Arbor, MI 48109-2200 USA

**Keywords:** Oral tolerance, Food allergy, Dietary antigen, Commensal antigen, GAPs, SAPs

## Abstract

**Purpose of Review:**

Growing interest has focused on the cellular and molecular mechanisms by which dietary antigens cross the intestinal epithelium and shape tolerance, sensitization, and allergic responses. This review summarizes the current understanding of antigen transport across the intestinal epithelium in steady state and food-allergic conditions.

**Recent Findings:**

Luminal antigens cross the intestinal epithelium via M cell-mediated transcytosis, transepithelial dendrites, paracellular leaks, receptor-mediated transcytosis, and goblet cell- and secretory antigen passages (GAPs & SAPs). These processes deliver antigens to the underlying immune compartment and drive tolerogenic or sensitizing immune responses. Recent studies have identified an association between dysregulated antigen transport mechanisms and development of food sensitization and reactivity, and these processes are regulated by cytokines and arachidonic acid-derived metabolites.

**Summary:**

Elucidating the mechanistic distinction between homeostatic and pathological antigen transport could identify novel targets that modulate antigen bioavailability to immune cells, potentially offering intervention strategies for food allergy prevention and treatment.

## Introduction

Gastrointestinal (GI) mucosal epithelia act as a physical barrier separating the external environment from the underlying tissue and immune compartment, to protect against exposures to harmful substances or pathogens [1]. On average, ~ 88.2 g of dietary protein is consumed by adults daily, making up 14% to 16% of the total caloric intake [2]. The diverse commensal microbiome and dietary proteins expose the mucosal epithelia of the GI tract to a vast array of antigenic epitopes [3]. To avoid the development of adverse immune reactions to harmless dietary protein, multiple preventative mechanisms have evolved, including (1) non-immunogenic degradation; (2) GI lumen retention; and (3) the development of oral tolerance, a local and systemic immune unresponsiveness [4].

Immune tolerance is the default response to GI luminal antigens [5, 6]. Immune unresponsiveness is achieved through the transport of dietary and commensal antigens across the selectively permeable GI epithelium and presentation of these antigens to the underlying immune compartment. The presentation of antigen to the immune compartment leads to the development of antigen-specific forkhead box p3-positive (Foxp3^+^) regulatory T (T_reg_) cells that suppress subsequent immune reactivity to these antigenic epitopes. The spatial and temporal localization of these tolerogenic processes within the GI compartment appears to be critical for the successful development of oral tolerance [5, 7–9]. Failure to develop tolerance to the commensal microbiome or dietary antigens can result in the development of GI diseases such as Crohn’s disease and ulcerative colitis, or food allergy and celiac disease, respectively [3, 6].

Several transport mechanisms for intact dietary antigen and commensal antigen across the GI epithelium have been described [10–17]. The intestinal epithelial cell (IEC) types involved, rate, and the spatial location of these uptake mechanisms are dependent on various factors, including antigen type and size, age, and sensitization status [5, 7–10, 12, 18]. Notably, dietary antigen uptake under food-sensitizing *or* allergic conditions differs from that in the non-allergic state, which has led to the concept that dysregulation of dietary antigen uptake promotes food sensitization and reactivity [12, 13, 18, 19]. However, critical questions like (1) whether an inherently altered antigen uptake landscape predisposes to sensitization or (2) whether sensitization drives secondary changes in uptake dynamics, and (3) what mechanisms regulate these changes, remain.

A deeper understanding of the role of dietary antigen transport mechanisms across the GI epithelium and the presentation of these antigens to the underlying immune compartment could lead to the identification of new clinical targets relevant for the treatment and prevention of inflammatory bowel diseases (IBD) and food allergies. This review focuses on studies that have expanded our understanding of how dietary antigens are translocated across the intestinal epithelium and presented to the small intestinal (SI) immune compartment, providing an overview of the differences present in health and disease.

## Oral Tolerance to Commensal and Dietary Antigens

The phenomenon of oral tolerance was first documented at the beginning of the 20th century through studies examining drug and food allergies, where prior feeding of corn or specific drugs to guinea pigs prevented allergic responses following subsequent active sensitization [20, 21]. This early work established that oral antigen exposure could induce protective immune unresponsiveness, a concept now formalized as oral tolerance. This concept describes the development of antigen-specific systemic immune unresponsiveness to orally ingested antigens [5, 6, 15, 20–22]. Within the GI tract, this discriminatory capacity exhibits distinct spatial organization based on antigen type [6, 23, 24]. Moreover, the SI serves as the primary site for dietary antigen tolerance, whereas the colon, which harbors the majority of the GI microbiome, functions as the critical compartment for establishing commensal tolerance [6, 23, 24].

To generate luminal antigen-specific immune responses, luminal antigens are presented to the mucosal immune compartment, located in the lamina propria (LP) or gut-associated lymphoid tissues (GALT), either through direct antigen sampling of the GI lumen or through transport of antigen across the GI epithelial barrier [6, 11, 14–17, 25]. The canonical view has been that GI CD103^+^ conventional dendritic cells (cDCs) are the critical antigen-presenting cells (APCs) that promote the tolerogenic response [6, 26]. GI CD103^+^ cDCs can be subdivided into cDC1 (CD103^+^CD11b^-^) and cDC2 (gut-specific CD103^+^CD11b^+^ and CD103^-^CD11b^+^) cells [27]. Experimental evidence has identified a role for both cDC1 and cDC2 subsets in the development of immune tolerance [6]. Transport of luminal antigen across the GI epithelium leads to CD103^+^ cDCs antigen uptake and trafficking of the DCs via the lymphatic system to the draining mesenteric lymph nodes (MLN) in a CC-motif chemokine receptor 7 (CCR7)-dependent manner [6, 25, 28, 29]. In the MLN, cDCs present antigen to naïve CD4^+^ T cells and promote the induction of antigen-specific FOXP3^+^ T_reg_ cells [6, 26, 28, 30–33]. GI CD103^+^ cDCs are thought to drive the induction of FOXP3^+^ T_reg_ cells through the vitamin A metabolite retinoic acid (RA) and TGFβ [6, 29, 32–34]. RA induces the expression of CCR9 and α4β7 on peripherally induced FOXP3^+^ regulatory T (pT_reg_) cells, which act as gut-homing molecules and direct the migration of activated FOXP3^+^ pT_reg_ cells to the GI tissue [6, 33]. In addition to inducing the formation of FOXP3^+^ pT_reg_ cells from naïve CD4^+^ T cells, CD103^+^ DCs also aid in the maintenance of existing FOXP3^+^ T_reg_ cell populations [32]. The T_reg_ cells of particular importance for the development of oral tolerance are extra-thymically induced FOXP3^+^ pT_reg_ cells [6, 30, 31]. The FOXP3^+^ pT_reg_ cells can be subdivided into retinoic acid receptor-related orphan receptor gamma transcription factor-expressing (RORγt^+^) FOXP3^+^ pT_reg_ cells, which drive tolerance to commensal microbes, and RORγt^−^ FOXP3^+^ pT_reg_ cells, which promote tolerance to dietary antigens [6]. These subsets of FOXP3^+^ pT_reg_ cells maintain a tolerant homeostatic environment predominantly through secretion of the cytokines TGF-β and IL-10 [35–38].

**Fig. 1:**
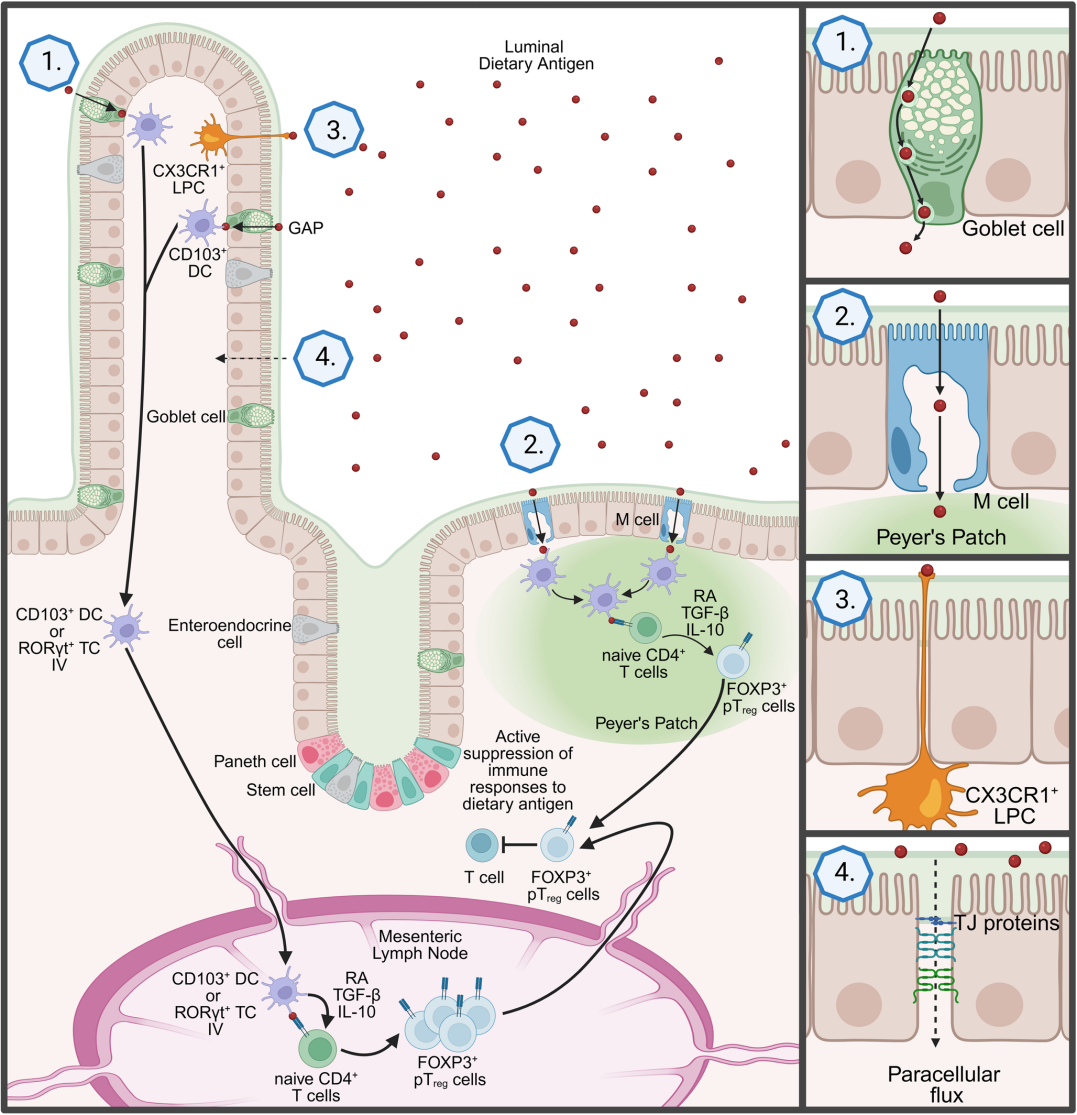
**Pathways of luminal antigen uptake across the intestinal epithelium to induce oral tolerance.** Luminal antigens (red) cross the intestinal epithelium through several routes: (1) transcellular transport via goblet cell antigen passages (GAPs), (2) transport through specialized microfold (M) cells overlying Peyer’s patches, and (3) direct sampling of luminal antigens by transepithelial dendrites from CX3CR1^+^ mononuclear lamina propria cells (LPC). (4) Tight junction (TJ) proteins prevent the uncontrolled uptake of luminal antigens through paracellular flux. After uptake, antigen is subsequently captured by CD103^+^ dendritic cells (DCs) or RORγt^+^ Thetis IV cells, which migrate to the mesenteric lymph node and promote the differentiation of naïve CD4^+^ T cells into FOXP3^+^ peripherally induced regulatory T (pT_reg_) cells via retinoic acid (RA), TGF-β, and IL-10. FOXP3^+^ pT_reg_ cells mediate active suppression of immune responses to dietary antigens, contributing to the maintenance of intestinal homeostasis.

In addition to cDCs, additional APCs have been linked to the development of tolerance [5, 39–41]. CX3CR1^+^ mononuclear lamina propria cells (LPCs) have been identified to play a role in the development of tolerance to the GI microbiota and dietary antigens. The production of IL-10 and antigen presentation by CX3CR1^+^ LPCs promote the formation of T_reg_ cells and reduce the load of T helper 1 (Th1) cells [39]. Furthermore, an alternative APC known as RORγt^+^ Thetis IV cells has been implicated in driving similar processes [5, 40, 41]. RORγt^+^ Thetis IV cells were originally characterized as an ILC3 subset, however recently been termed either Janus or Thetis cells [6]. In the recent study by Cabric et al., RORγt^+^ Thetis IV cells were shown to be involved in the generation of commensal and dietary antigen-specific pT_reg_ cells [5, 40, 41]. RORγt^+^ Thetis IV cells are an APC type almost exclusively found in the MLN [40–42]. These cells take up luminal antigens and migrate to the draining lymph nodes, where they stimulate the generation of antigen-specific T_reg_ cells from naïve CD4^+^ T cells [40, 41]. The differentiation into T_reg_ cells is mediated by antigen presentation through the major histocompatibility complex class II (MHCII), as well as the conversion of pro-TGF-β into active TGF-β through the integrin α_v_β_8_ expressed on these RORγt^+^ Thetis IV cells (Fig. 1) [6, 40–42].

Several studies have demonstrated that early life is a critical period in the development of oral tolerance [6, 40, 41, 43–46]. The weaning period is thought to be the initial period when the GI immune system of infants first encounters both dietary and microbial antigens, and it is during this period that the host must recognize these antigens as harmless to prevent the later development of adverse immune reactions to these antigens [42]. Interestingly, during the periweaning phase, the number of Thetis IV cells is increased compared to later in life [40–42]. Furthermore, during oral antigen exposure, T_reg_ cell differentiation is enhanced in early-life compared to adult mice [8, 41]. The timeframe of weaning has also been associated with a transient inflammation and a subsequent expansion of the T_reg_ cell population to manage the reaction to the expanding microbiome [6].

## Biological Mechanisms of Food Allergy

Atopic dermatitis and other skin-damaging comorbidities that exhibit a compromised cutaneous epithelial barrier have been linked to the development of food allergy [35, 47–50]. Consistent with this, the severity of skin damage positively correlates with the risk of developing food allergies [35]. These clinical data have led to the concept that cutaneous barrier disruption facilitates dietary antigen penetration and subsequent sensitization [19, 35, 48, 49, 51].

**Fig. 2:**
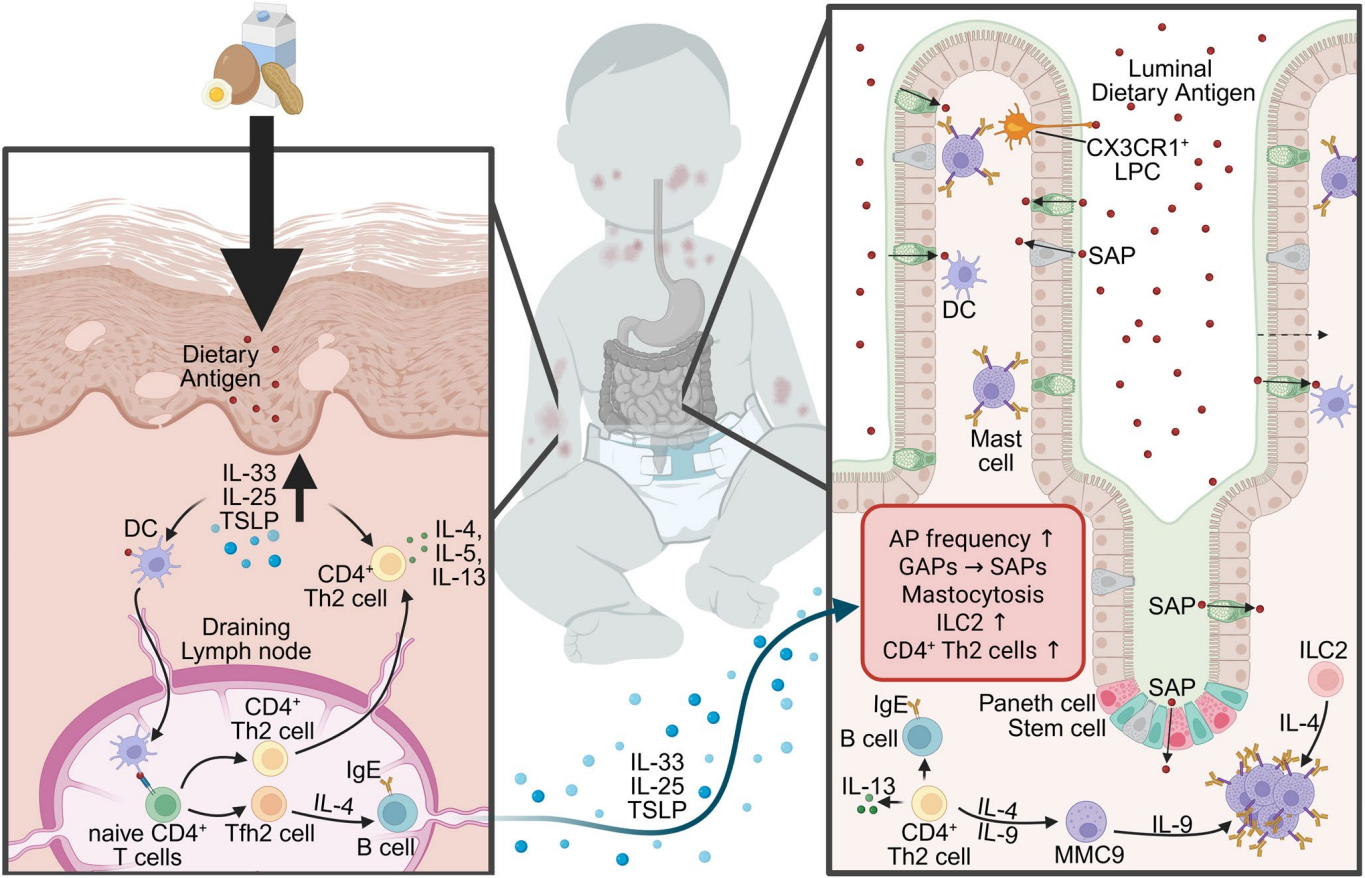
**Early-life skin barrier dysfunction promotes allergic gut tropism through Th2-driven immune pathways.** Disruption of the skin barrier (left) enables dietary antigen entry to the skin and induces epithelial-derived pro-type 2 alarmins IL-33, IL-25, and TSLP, which promote Th2 differentiation and IgE class switching in draining lymph nodes. During subsequent oral dietary antigen exposures (right), these Th2 responses and cytokine signals drive allergic gut tropism, characterized by increased antigen passage (AP) frequency, conversion of goblet cell antigen passages (GAPs) to secretory antigen passages (SAPs), mastocytosis, and expansion of ILC2s and CD4^+^ Th2 cells. Enhanced IL-4, IL-5, IL-9, and IL-13 production further amplifies IgE-mediated mast cell activation and the emergence of IL-9^+^ mucosal mast cells (MMC9), linking cutaneous sensitization to intestinal allergic inflammation in early life

**Fig. 3:**
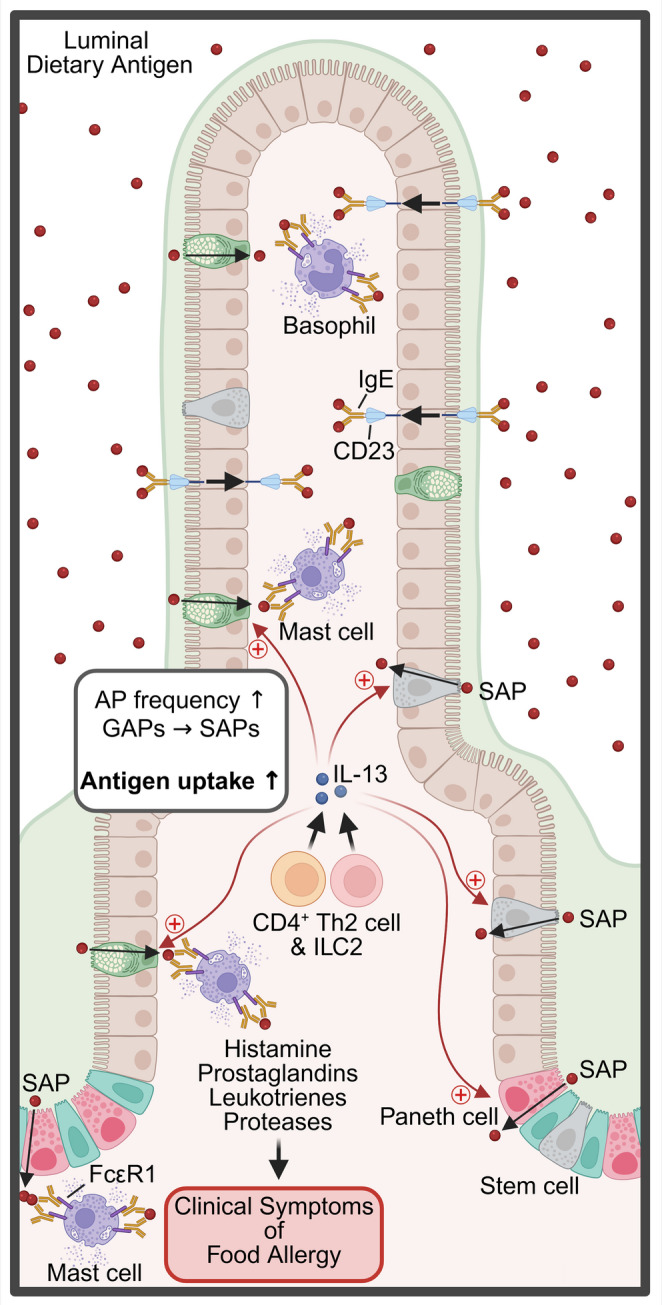
**Dysregulated antigen uptake and immune responses in food allergy.** In food-allergic conditions, dietary antigens (red) cross the intestinal epithelium through alternative and enhanced routes. Transcytosis via secretory antigen passages (SAPs) through goblet cells, enteroendocrine cells, and Paneth cells, and binding of luminal antigen-IgE immune complexes to epithelial CD23 (FcεRII), which facilitates antigen transcytosis. The shift in antigen passage landscape, combined with increased passage frequency, enhances antigen transport across the intestinal epithelium . Th2-derived cytokines such as IL-13 drive the switch from the tolerogenic GAPs to the pro-allergic SAPs. Upon exposure to dietary antigen, cross-linking of FcεRI-bound IgE on mast cells triggers the release of histamine, prostaglandins, leukotrienes, and proteases, leading to the clinical symptoms of food allergy.

The exposure of the antigens to damaged skin leads to the production of the pro-type 2 alarmins IL-25, IL-33, and thymic stromal lymphopoietin (TSLP) [15, 52–58]. The pro-type 2 alarmins are thought to prime local DCs to take up the dietary antigen and home to the draining lymph nodes, where they promote activation and differentiation of CD4^+^ T cells into antigen-specific T helper 2 (Th2) cells and T follicular helper cells [22, 35, 59, 60]. Th2 cells secrete cytokines like IL-4, IL-5, and IL-13 [22, 61]. IL-4 is required for class switching of B cells to produce antigen-specific IgE antibodies [62]. The skin-derived dietary antigen-specific CD4^+^ Th2 and IgE response is recruited to the SI in a process known as *allergic gut tropism* [35]. This reshaping of the SI immune microenvironment primes the GI tract to react to subsequent oral dietary antigen exposures [35]. CD4^+^ Th2 cells and IL-4 drive the production of IL-9 by hypo-granular mucosal mast cells (MMC9s) [63–66]. These IL-9/IL-9R signals support further MMC9 expansion and intestinal mastocytosis (Fig. 2) [63–67]. Subsequent oral exposure to the dietary antigen leads to crosslinking of IgE/Fcε-receptors on mast cells and basophils and their degranulation, leading to the release of autacoid mediators (histamine, leukotrienes, tumor necrosis factor (TNF)-α, chymase, and serotonin) (Fig. 3) [12, 15, 22, 35, 54, 56–58, 62, 68–70]. The released mediators act on the GI tract, the cutaneous, respiratory, neurological, and cardiovascular system, driving the clinical manifestations of a food allergic reaction [12, 15, 35, 68]. Histamine, platelet-activating factor (PAF), serotonin, and leukotriene C4 (LTC4) drive fluid extravasation, leading to increased hemoconcentration and hypovolemic shock [35, 71]. Histamine promotes concentration-dependent vasodilation and shock [72]. In addition, histamine has been shown to decrease respiratory function and body temperature [73, 74]. Similarly, mast cell-derived chymase has also been linked to the hypothermic response by activating the thermoregulatory neural circuit [35, 75]. Whilst serotonin and PAF synergistically induce allergic diarrhea [35, 76].

## Transport of Luminal Antigen across the Epithelium

The GI epithelium forms a barrier between the luminal contents, including dietary and commensal antigens, and the mucosal immune compartment located under the epithelial layer [1]. This GI epithelium is made up of a single layer of six major differentiated epithelial cell types: absorptive enterocytes, or colonocytes in the colon, secretory cells (enteroendocrine cells, Paneth cells, goblet cells, and tuft cells), and microfold (M) cells, with enterocytes being the most common cell type of the GI epithelium [10, 77–79].

Food is made up of several macronutrients: carbohydrates, fats, and proteins. Through digestion, these macronutrients are broken down and taken up by the body to supply nutrients required for energy and survival [80]. During digestion, food proteins are broken down into individual amino acids and small polypeptides (Di- and Tripeptides), which are transported from the SI lumen through the absorptive enterocytes to the portal vein and are used as building blocks for protein synthesis by the body [81, 82]. Partially or undigested dietary and commensal proteins, with antigenic potential, also cross the GI epithelium [10–17]. Several mechanisms have been identified that transport intact dietary antigen across the epithelium, like M cell-mediated transcytosis, transepithelial dendrites (TEDs), paracellular leaks, canonical goblet cell antigen passages (GAPs), and non-canonical secretory antigen passages (SAPs) [12, 19]. These distinct uptake routes are thought to direct the induction of either immune unresponsiveness or reactivity to these antigens.

### TEDs

DCs can present antigen to other immune cells and thereby prime the immune system to become tolerant to self-antigen or mount an immune reaction to invading pathogens [16]. A subgroup of CX3CR1^+^ DCs has been described to directly sample luminal antigen in the intestine, using CX3CR1-dependent projections called transepithelial dendrites (TEDs) [16, 17]. Subsequent reports demonstrated CX3CR1^+^ tissue resident macrophages can also act in a similar manner, therefore coining the term CX3CR1^+^ mononuclear lamina propria cells (LPCs) (Fig. 1) [10, 29, 83]. CX3CR1^+^ LPCs extend TEDs into and through the paracellular spaces of IECs [16, 17, 84]. These projections form mono- and multi-ball-like branches, which serve as a point of contact for luminal antigens [16, 85]. CX3CR1^+^ LPCs are known to express tight junction (TJ) proteins (occludin, claudin 1, E-cadherin, β-catenin, junctional adhesion molecule (JAM)). The extended dendrite TJ proteins interact with intestinal epithelial TJ proteins, resealing the paracellular space and maintaining intestinal epithelial barrier integrity [17, 84].

The primary purpose of TEDs remains unclear. CX3CR1^+^ LPCs that form TEDs are restricted to the SI villi [16, 83, 85]. A subset of lysosome-expressing DCs located in the subepithelial dome of Peyer’s patches has been described to extend dendrites through transcellular pores in M cells to sample luminal antigens [10, 86]. TEDs have been shown to be activated by pathogens (e.g., *Salmonella typhimurium*) and promote bacterial antigen presentation to the immune compartment [11, 85, 87]. However, luminal bacterial antigen uptake in mice lacking TEDs (*CX3CR1*^GFP^ C57BL/6) is not impaired [87], suggesting the presence of alternative uptake mechanisms for luminal pathogens [87]. Consistent with this, BALB/c mice have been reported not to possess TEDs, and pathogen (*Aspergillus conidia* and *Salmonella typhimurium*) uptake was not impaired [87]. A recent study demonstrated that in humans, the formation of TEDs by cDC1 cells is mediated by GPR31, which is expressed on 35% of cDC1 cells [88]. The luminal metabolite pyruvate produced by GI microbes was shown to activate GPR31 expressed on cDC1 cells, inducing TED formation [88].

Studies using ex vivo imaging techniques have revealed that removal of mucus and luminal contents to image TEDs is sufficient to induce the formation of TEDs in the SI [16, 17, 84, 89]. In studies where luminal contents and mucus remain undisturbed (in vivo imaging techniques), LPCs within the GI tissue extended their TEDs into the intercellular spaces of the epithelium, but not into the GI lumen [7, 11]. Extension of TEDs into the SI lumen required a bacterial (*Salmonella typhimurium*) challenge [11]. In a more recent study, in vivo imaging captured rare events of TED extension into the lumen of the distal SI, but remained absent in the proximal SI and colon [25].

To our knowledge, TEDs have not previously been described as playing a role in dietary antigen oral tolerance or the onset of a food allergic reaction. The demonstration that TEDs are primarily restricted to the distal SI and are CX3CR1-dependent suggests a potential role for TEDs in sampling luminal food antigens and dietary antigen tolerance [11, 16, 25, 34]. However, certain mouse strains (e.g., BALB/c) lack TEDs entirely but mount normal tolerogenic immune responses to dietary antigens [34, 87], indicating that TEDs are dispensable for oral tolerance induction. The impact of food allergic Th2 responses on TEDs also remains largely unexplored, though IL-4 downregulated CX3CR1 expression on LP phagocytes [87, 90], suggesting that allergic inflammation would suppress rather than promote TED formation.

### M Cell-Mediated Transcytosis

M cells are a subtype of absorptive IECs with a strong link to the immune compartment. They are commonly located in the epithelium above Peyer’s patches, cecal patches, and isolated lymphoid follicles [6, 15]. M cells are involved in the uptake of luminal antigen and transport to the underlying immune cells (Fig. 1) [14, 15]. Transport of Gl luminal antigens through M cells to the underlying immune compartment is mediated via clathrin-coated endocytic and pinocytic mechanisms [91–94]. M cells possess invaginated folds within the basolateral membrane that create a pocket known as the lymphocyte-microfold cell (lympho-M) pocket. The Lympho-M pocket permits the strategic positioning of immune cells, including DCs, B and T cells, and facilitates the efficient transfer of luminal antigens to the underlying immune cells [95]. M cell-mediated transcytosis is thought to be involved in the induction of oral tolerance to dietary antigens [96]. Indeed, M cell-mediated uptake of luminal ovalbumin (OVA) decreased the abundance of the OVA-specific antibodies IgG and IgA and CD4^+^ T effector cell responses while increasing the number of tolerogenic FOXP3^+^CD4^+^ T cells in the gut LP [96]. Furthermore, oral OVA administration in mice with disrupted Peyer’s patches was associated with splenic CD4^+^ T cell proliferation and delayed-type hypersensitivity responses after systemic immunization [97]. However, mice deficient in Peyer’s patches with a dysregulated M cell function, or mice deficient in M cells, such as RANKL-deficient mice and mice with intestinal epithelial conditional deletion of Tumor necrosis factor receptor (TNFR)-associated factor 6 (TRAF6), generate tolerogenic responses to dietary antigens. These observations suggest that M cell-dependent dietary antigen presentation is not required for the oral tolerance response but may contribute to the development of tolerance [98–101]. The role of M cell-mediated luminal dietary antigen uptake in food allergic conditions remains elusive.

### Antigen Transport Across Secretory Cells: GAPs and SAPs

**Fig. 4:**
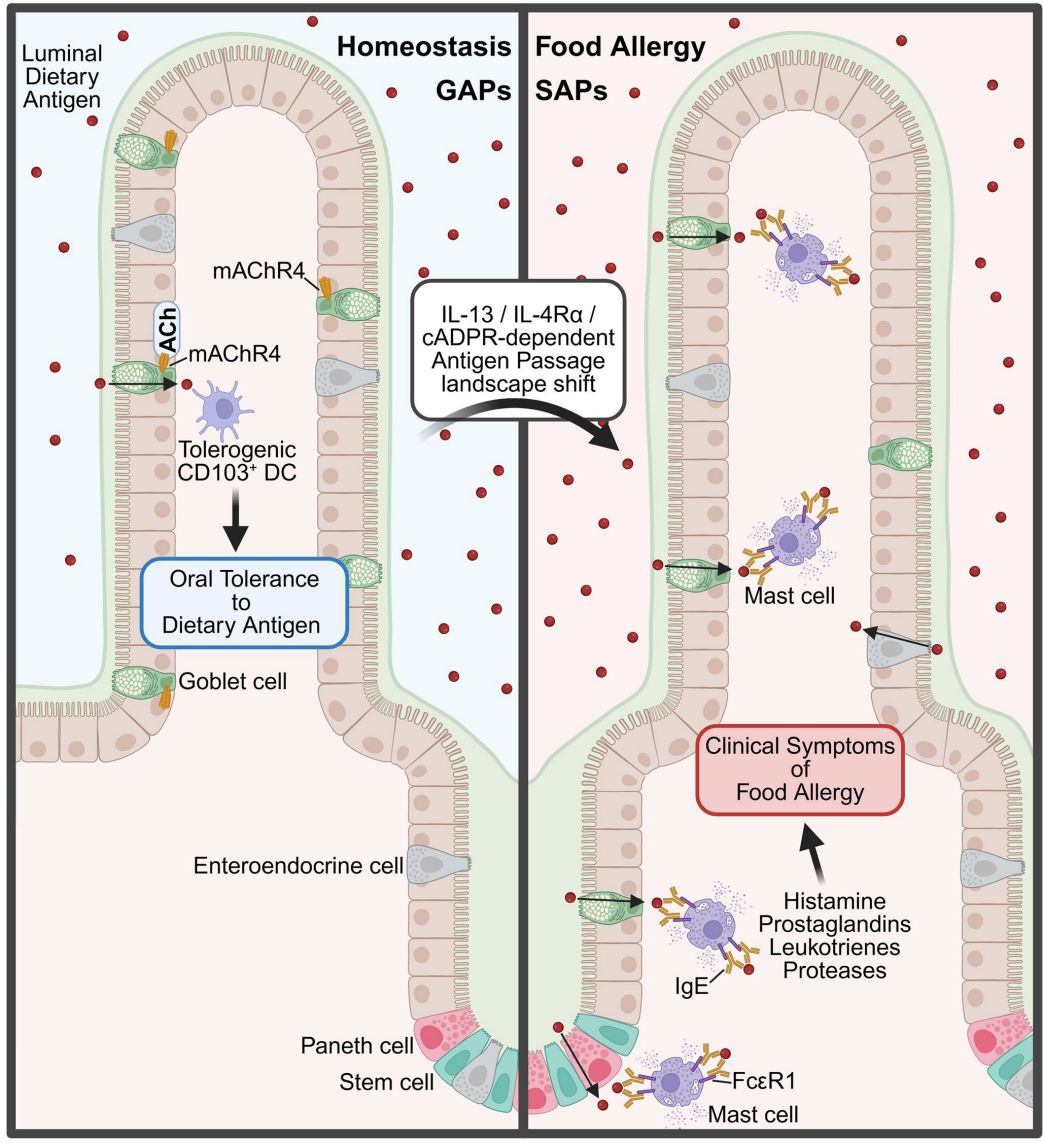
**Antigen passage landscape at homeostasis vs. during food allergic conditions.** During homeostasis, dietary antigen is transported across goblet cells to underlying tolerogenic CD103^+^ dendritic cells (DCs), in a process called goblet cell antigen passages (GAPs). This transport is activated through the binding of acetylcholine (ACh) to the muscarinic acetylcholine receptor 4 (mAChR4). In food allergic conditions, this antigen passage landscape shifts from tolerogenic GAPs to pro-allergic secretory antigen passages (SAPs). SAPs are formed in an IL-13/IL-4Rα/cADPR-dependent manner, leading to an increased antigen transport through goblet cells, enteroendocrine cells, and Paneth cells to underlying mucosal mast cells. Crosslinking of the FcεR1 on mast cells leads to the release of autacoid mediators (histamine, prostaglandins, leukotrienes, and proteases) that mediate the clinical symptoms of food allergy

Goblet cells are the most abundant secretory cells located in the GI epithelium [15, 77]. These cells are best known for their role in mucin production and secretion [9, 15, 77]. These mucins form the mucus layer that aids the movement of luminal contents and forms a physical barrier to protect against luminal pathogens [15, 77]. Goblet cells have also been shown to transport luminal protein antigens across the GI epithelium and present these antigens to underlying LP-DCs in a process called goblet cell antigen passages (GAPs) (Table 1) (Fig. 4) [11, 12].

**Table 1:** Comparison of antigen passages in small intestinal epithelium

	GAPs	SAPs	References
State	Homeostasis	Food Allergy	[11, 12, 25]
**Cell type**	Goblet cells	Goblet cells, Enteroendocrine cells, and Paneth cells	[11, 12]
**Localization in the villus/crypt axis**	Villus	Villi and Crypt	[11, 12]
**Frequency**	~ 0.5 APs per villus/crypt axis	~ 4.5 APs per villus/crypt axis	[12]
**Mechanism of Induction**	Muscarinic acetylcholine receptors 1 and 4	IL-13/IL-4Rα/cADPR-dependent	[12, 102]
**Transport target in the lamina propria**	CD103^+^ Dendritic cells	Mucosal Mast cells	[11, 12]

GAPs are present in all segments of the naïve SI (duodenum, jejunum, ileum) [11], and the distal part of the colon (distal descending colon and sigmoid colon) [12, 15, 25]. Luminal antigen transport through GAPs has been described in the SI of both mice and humans [11]. GAP formation is dependent on acetylcholine (Ach) signaling and the downstream release of intracellular calcium (Ca^2+^) stores [102, 103]. Whilst GAP formation in the SI is activated through the muscarinic Ach receptor 1 (mAChR1) and mAChR4, mAChR1 and mAChR3 regulate the mechanism in the distal colon [12, 102]. After activation of GAPs, luminal contents are taken up into the goblet cell through fluid-phase endocytosis [102]. In contrast to these steady-state functions of GAPs, SI GAP formation is inhibited by pathogens such as *Salmonella* [104]. The blockage of luminal antigen transport through GAPs is mediated through *Salmonella*-induced IL-1β, which activates the IL1R-MYD88-pathway, stimulating epidermal growth factor receptor (EGFR)-dependent inhibition of SI GAPs [104]. It is speculated that inhibition of SI GAPs by pathogenic bacteria is a host-dependent response to prevent systemic dissemination. Similarly, in the adult proximal colon, mAChR-dependent GAP formation is prevented by Myd88 activation through the colonic microbiota and subsequent signaling through EGFR and p42/p44 MAPK [103].

The most common LP DC subtype that takes up SI dietary antigen from GAPs is CD103^+^ DCs [11]. CD103^+^ DCs are known to influence regulatory T cell development [11]. In contrast, colonic GAPs predominantly transport microbes and macromolecules to CX3CR1^+^ DCs to induce antigen-specific tolerance to gut bacteria [7]. Consistently, the presence of GAPs has been linked to the induction of food-specific T_reg_ cell responses and the establishment of oral tolerance [7, 8, 15]. In addition to the role in transporting antigen to induce oral tolerance, GAPs have also been implicated in regulating the production of the pro-tolerogenic IL-10 by CD11c^+^MHCII^+^CD11b^+^F4/80^+^ LP-APC [25], suggesting additional pathways in which GAPs facilitate the development of oral tolerance.

As described in a previous section of this review, several studies have demonstrated that early life is a critical period in the development of oral tolerance [6, 40, 41, 43–46]. In addition to the periweaning window described above, a specific preweaning interval has been identified that is linked to an antigen passage-mediated development of antigen-specific tolerance to the colonic microbiome and food allergens [7, 8]. In this preweaning interval, colonic luminal antigen was demonstrated to be transported across the epithelium through GAPs [7, 8]. Specifically, GAPs were demonstrated to be absent in the GI tract until day of life (DOL) 10, at which point they were reported to form in the colon [7, 8]. Between DOL10 to DOL20, the level of GAPs in the colon remains constant and then decreases after DOL20. Weaning and commensal microbiota colonization after DOL21 of the proximal colon leads to inhibition of GAPs via TLR/MYD88-dependent transactivation of the EGFR pathway [7, 105]. In the SI, GAPs appear around DOL12 and are retained throughout adulthood [7, 8]. This switch in the spatial localization of antigen transport is regulated through the presence of EGF in maternal breastmilk, which inhibits GAP formation in the proximal GI tract [7, 8]. The prenatal uptake of luminal antigens through colonic GAPs is thought to promote tolerance to colonic microbes and dietary antigens [7, 8]. Notably, disruption of the spatial and temporal regulation of GAP function has been shown to disrupt colonic pT_reg_ development and promote a skewing of a sensitizing dietary antigen immune response. Moreover, disruption of chronological GAP patterning by desynchronizing dams and pups led to diminished dietary antigen colonic pT_reg_ development and formation of an unrestrained CD4^+^ Th2 response [8].

While goblet cells are the dominant secretory cell involved in antigen passages at steady state, evidence suggests possible involvement of Paneth cells and enteroendocrine cells [25]. Antigen transport through Paneth cells has been described in all sections of the SI. However, transport through enteroendocrine cells is restricted to the duodenum [25]. Currently, we do not understand the individual contribution of these secretory cell lineages to the sensitizing and tolerogenic immune responses [25, 102]. The demonstration that GAPs are ~ 1000-fold more common than TEDs and that APC populations isolated from mice lacking GAPs are unable to induce antigen-specific CD4^+^ T cell responses, indicates that GAPs are critical for delivering luminal antigens to APCs for subsequent T cell priming. The demonstration that GAP inhibition decreased systemic tolerance to dietary antigens suggests that GAPs prime for tolerogenic responses [25].

We recently demonstrated that a gain-of-function mutation in the Il4rα (Il4rα^F709^ mice) and enhanced IL4Rα signaling is sufficient to promote a shift in the antigen-passage-landscape within the SI, with SI MUC2^+^ cells (Goblet cells) and MUC2^−^ cells (enteroendocrine and Paneth cells) participating in dietary antigen uptake. This process is referred to as secretory antigen passages (SAPs) (Table 1) [19]. Activation of the SAPs and oral exposure to OVA led to increased oral food sensitization and clinical reactivity [12]. Consistent with these observations, we demonstrated that SI SAPs transport dietary antigen from the apical to the basolateral side of the epithelium, where they interact with FcεRI^+^ c-Kit^+^ ST2^high^ mucosal mast cells (Fig. 4) [12]. Unlike steady state GAPs [105], SAPs are predominantly regulated by the Th2 cytokine IL-13, via a direct IL-4Rα-STAT6-independent PI3K-CD38-Cyclic ADP-ribose (cADPR)-dependent process [12]. Pharmacological and genetic blockade of IL-4Rα signaling on secretory IECs inhibited IL-13–driven SAP formation and protected against a food-induced anaphylactic reaction. These data suggest that GI-epithelial-dependent processes, such as SAPs, are important in the induction of the effector phase of food allergic reactions [12]. Importantly, GAPs and SAPs are conserved in human tissue and are regulated via m4AchR-dependent and IL-13–induced CD38/cADPR-dependent processes [12]. Taken together, these data suggest that the food-sensitized state and heightened levels of type 2 cytokines are sufficient for reprogramming intestinal epithelial antigen passages and presentation of dietary antigens to different immune compartments and cell populations.

### Paracellular Transport

The spaces between cells in an epithelium are connected by molecules called desmosomes, adherens junctions, and TJ proteins (occludins and claudins) [10, 106]. The selective barrier that is formed through these molecules prevents uncontrolled uptake of luminal macromolecules through the intracellular space between IECs, like dietary antigens, and microorganisms, like commensal or pathological microbes (Fig. 1) [106].

Inflammation or immune molecules have been shown to alter paracellular transport and permit transport of antigen across paracellular spaces [107–118]. Inflammatory cytokines, like interferon-γ (IFNγ) and TNF, have both been linked to an increased paracellular leakiness, due to myosin II regulatory light chain (MLC) phosphorylation-dependent changes in expression levels of several TJ proteins, including occludin and claudin-1 [108–111]. Similarly, basolateral exposure to the cytokines IL-4 and IL-13 has also been linked to decreased transepithelial resistance and increased paracellular flux due to perturbation of TJ proteins [107, 112, 113]. IL-13 specifically has been linked to an increased number of apoptotic IECs and expression of the pore-forming TJ protein claudin-2 [118]. Additional external factors capable of altering TJ proteins and thereby paracellular flux are IL-22, chymase, tryptase, and eosinophil-derived granule proteins [114–117]. Paracellular leak is unlikely to play a role in steady-state luminal antigen delivery to the LP immune compartment, as LP-DCs derived from Goblet cell-deficient mice were unable to induce antigen-specific T cell signaling, even though paracellular leak was detected [11]. In addition, a mouse model lacking GAPs demonstrated an antigen size-dependent increased uptake of luminal antigen through paracellular spaces, whilst SI APCs demonstrated lower levels of antigen [25]. This suggests that paracellular leak is not linked with luminal antigen loading on GI APCs.

However, some studies support that altered paracellular permeability can promote dietary antigen responses [67, 119–126]. Mast cells have been demonstrated to mediate epithelial permeability through the degradation of the TJ protein occludin [67, 127], allowing for the paracellular translocation of dietary antigens across the GI epithelium [67]. This mast cell-linked increased translocation of luminal antigens has been associated with the induction of dietary antigen sensitization [67]. Furthermore, some possible environmental exposures have been linked with disrupted paracellular transport and a risk for dietary antigen sensitization [119–121]. A study with rat intestinal tissue demonstrated that exposure to cholera toxin, a common adjuvant in food allergy models, increases the level of claudin-2, decreases the level of tricellulin, reduces transepithelial resistance, and widens intercellular spaces between IECs [119]. Consistent with these findings, studies have demonstrated that oral exposure to cholera toxin increases the luminal uptake of dietary antigens and counteracts tolerogenic responses to oral antigen exposures [122, 123]. Furthermore, zonula occludens toxin has been shown to alter the structure of paracellular tight junctions [121]. Exposure of the agonist AT-1002 leads to increased permeability and promotes SI IgE production, preventing the development of oral tolerance after oral OVA exposure [120]. These studies demonstrate that increased dietary antigen uptake in the GI tract due to a damaged intestinal epithelium may be a risk factor for the development of food allergies. This concept is supported by multiple clinical studies demonstrating an increased intestinal permeability in food-allergic infants and adults [124–126].

### LTD_4_ Promotes Antigen Transport

A recent study in mice identified that the localization and enzymatic activity of the membrane-bound dipeptidase (DPEP1) in IECs is linked to altered dietary antigen transport in food allergy susceptible mice [18]. DPEP1 catabolizes the arachidonic acid-derived cysteinyl leukotriene (CysLT) leukotriene D_4_ (LTD_4_) into leukotriene E_4_ (LTE_4_) [18, 128]. CysLTs are a group of inflammatory mediators involved in the chemoattraction of mast cells and eosinophils, vascular permeability, smooth muscle contraction, and mucus secretion [128–132]. These mediators are primarily produced by leukocytes, including mast cells [128, 131]. In the context of food allergy, CysLTs have been primarily described to induce the symptoms of anaphylaxis [62, 128].

Hoyt et al. demonstrated that mouse strains that are resistant to the development of food allergy, such as C57BL/6 mice, transport less dietary antigen across the GI epithelium compared to food allergy susceptible mouse strains, such as C3H/HeJ mice [18]. These data suggest that baseline differences in dietary antigen passage in the GI tract may predispose to the development of food allergy. Increased luminal antigen uptake was associated with an increased number of GAPs in the villus of C3H/HeJ mice in comparison to C57BL/6 mice [18]. Notably, C3H/HeJ mice demonstrated some antigen transport across the epithelium that was independent of goblet cells (WGA^−^ cells) [18]. Mechanistic studies revealed that the dietary antigen transport across the GI epithelium in C57BL/6 mice could be increased through blocking the catalyzation of LTD_4_ or oral administration of LTD_4_ [18]. Consistent with this, treatment with zileuton, which ablates all CysLT production, decreases luminal dietary antigen uptake and inhibits clinical reactivity in food allergy susceptible mice [18]. The reduced enzymatic activity of DPEP1 in the food allergy susceptible C3H/HeJ mice, in combination with the increased level of LTD_4_ and rate of dietary antigen transport across the intestinal epithelium, offers an insight into genetic differences that could predispose to the development of food allergy [18].

### Receptor-Mediated Transport

IgE is essential for the allergic immune response to dietary antigens [58]. The high-affinity receptor FcεRI and low-affinity receptor FcεRII (CD23) are the primary IgE receptors [133]. In contrast to FcεRI, the role of CD23 in allergic disease appears to be complex and can positively and negatively regulate IgE production [133]. CD23 has been described to be expressed on IECs, as well as B cells, T cells, DCs, monocytes, neutrophils, eosinophils, and respiratory epithelial cells [133, 134]. Previous studies have demonstrated that CD23 is also expressed on the apical membrane of enterocytes [13, 135]. This expression is described to increase after sensitization to dietary antigens [135]. This is likely due to the presence of IL-4 which leads to an upregulation of epithelial CD23 [136]. Similarly, food challenges lead to increases in soluble CD23 and antigen-specific IgE in stool [13]. CD23 has been implicated in the specific transepithelial transport of sensitized dietary antigen through enterocytes [13, 135]. Transcytosed dietary antigen-IgE complexes can induce degranulation in rat basophil leukemia cells [13]. These reports indicated that antigen-IgE immune complexes can be shuttled across the epithelium at mucosal surfaces (Fig. 3).

## Conclusion

The GI epithelium serves as a critical interface between the external antigenic environment and the underlying immune compartment, with its capacity to regulate luminal antigen transport playing a central role in determining whether tolerance or sensitization develops to dietary proteins. Under homeostatic conditions, multiple mechanisms, including M cell-mediated transcytosis, TEDs, and GAPs, facilitate the controlled sampling of luminal antigens and the induction of a tolerogenic immune response. The induction of oral tolerance is critically dependent on the precise spatial and temporal regulation of antigen uptake, particularly during early life developmental windows when the immune system first encounters dietary and microbial antigens. However, this process can be disrupted in food allergic conditions, where heightened type 2 inflammation fundamentally reshapes the antigen passage landscape through mechanisms such as IL-13-driven SAPs, ultimately redirecting dietary antigens to effector immune cells like mucosal mast cells rather than tolerogenic APCs.

A deeper mechanistic understanding of how dietary antigen transport is regulated and dysregulated holds significant promise for developing novel therapeutic and preventive strategies for food allergy and other immune-mediated gastrointestinal disorders. Emerging evidence suggests that genetic factors, such as variations in DPEP1 activity and consequent alterations in leukotriene D4 metabolism, may predispose certain individuals to enhanced antigen uptake even prior to sensitization, raising important questions about whether altered epithelial transport represents a primary risk factor or a consequence of established type 2 immunity. Critical knowledge gaps remain, particularly regarding how developmental programming of antigen transport mechanisms during the periweaning period influences long-term food allergy susceptibility, and how environmental factors and the microbiome interface with epithelial transport pathways to modify disease risk. Addressing these questions will be essential for translating our growing understanding of intestinal antigen handling into effective clinical strategies to prevent and treat food allergies.

## Key References

- McDole JR, Wheeler LW, McDonald KG, et al. Goblet cells deliver luminal antigen to CD103+ dendritic cells in the small intestine. Nature 483, 345-349, 10.1038/nature10863 (2012).
  - ○ In this first description of the goblet cell-associated antigen passages (GAP), the authors demonstrated the dynamic transport of SI luminal antigen across goblet cells after secretion of mucus. The transported antigen is described to be taken up by the tolerogenic LP CD103+ DCs. This SI-specific transport is identified as the primary antigen uptake mechanism at homeostasis.
- Noah TK, Knoop KA, McDonald KG, et al. IL-13-induced intestinal secretory epithelial cell antigen passages are required for IgE-mediated food-induced anaphylaxis. J Allergy Clin Immunol 144, 1058-1073 e1053, 10.1016/j.jaci.2019.04.030 (2019).
  - ○ We demonstrated an alteration in the SI antigen passage landscape in a food-allergic state. Clinical dietary antigens are described to be transported across not only goblet cells, but also enteroendocrine cells and Paneth cells during food allergy, across so-called secretory antigen passages (SAPs). SAPs were demonstrated to transport luminal antigens to underlying mucosal mast cells. This transport was shown to be mediated in an IL-13-CD38 cADPR-dependent pathway. Blockage of the IL-13 signaling attenuated both antigen transport and clinical anaphylaxis symptoms.
- Hoyt LR, Liu E, Olson EC, et al. Cysteinyl leukotrienes stimulate gut absorption of food allergens to promote anaphylaxis in mice. Science 389 (6760), 10.1126/science.adp0240 (2025).
  - ○ The introduction of the cysteinyl leukotriene LTD4 as a major regulator of the rate of dietary antigen transport at homeostasis demonstrates a novel insight into how genetic variances of the enzyme DPEP1 might predispose to the development of food allergy. The study demonstrates the ability to limit antigen uptake through treatment with the FDA-approved drug zileuton, which was associated with attenuation of anaphylaxis symptoms in mice.

## Data Availability

No datasets were generated or analysed during the current study.
